# Synthesis, Characterization, and BSA Binding Studies of Some New Benzamides Related to Schiff Base

**DOI:** 10.1155/2013/791591

**Published:** 2013-04-07

**Authors:** M. K. Prashanth, M. Madaiah, H. D. Revanasiddappa, K. N. Amruthesh

**Affiliations:** ^1^Department of Chemistry, University of Mysore, Manasagangotri, Mysore, Karnataka 570006, India; ^2^Department of Botany, University of Mysore, Manasagangotri, Mysore, Karnataka 570006, India

## Abstract

Condensation of amine **1** with aldehyde **2** gives Schiff base, * N*-(4-((benzofuran-2-ylmethylene) amino)phenyl)acetamide **3**. Schiff base on *N*-acylation with different substituted acid chlorides in the presence of triethylamine gives the corresponding benzamides, *N*-acetyl-*N*-(4-((benzofuran-2-ylmethylene)amino)phenyl)substitutedbenzamide (NABP) **5a–j**. The structures of newly synthesized compounds were characterized by elemental analysis, ^1^H NMR, ^13^C NMR FT-IR, and mass spectral studies. Compounds **3** and **5a–j** have been screened for their antimicrobial activity using the disc diffusion and minimum inhibitory concentration (MIC) method against the selected bacterial and fungal strain. Compounds **5a**, **5e**, **5g**, and **5h** were found to be more active against all tested strains. The antioxidant properties were evaluated by 2,2-diphenyl-1-picrylhydrazyl (DPPH) and superoxide radical scavenging methods. Compounds **5i** and **5j** showed predominant antioxidant activities among the synthesized analogues. The interaction between NABP and bovine serum albumin (BSA) was investigated using fluorescence and ultraviolet spectroscopic techniques at 298 K under imitated physiological conditions. The results revealed that NABP caused the fluorescence quenching of BSA through a static quenching procedure. The binding constants and the number of binding sites were calculated. The binding distance between the donor (BSA) and acceptor (NABP) was determined based on Forster's theory.

## 1. Introduction

Schiff bases represent an important class of organic compounds, especially in the medicinal and pharmaceutical field. Schiff bases appear to be important intermediates in a number of enzymatic reactions involving interaction of the amino group of an enzyme, usually that of a lysine residue, with a carbonyl group of the substrate [1]. Schiff bases, derived mostly from variety of heterocyclic rings, were reported to possess a broad spectrum and a wide variety of biological activities including antimicrobial, anti-inflammatory, anticonvulsant, antitumor, and anti-HIV activities [2–4]. It is evident that in azomethine derivatives the –HC=N linkage is an essential structural requirement for biological activity. The Schiff bases have emerged as antimicrobial agents of an immense interest because of their broad spectrum of *in*-*vitro* activity and their *in vivo* chemotherapeutic activity. In addition, benzamide derivatives which are the possible metabolites of benzoxazoles show various types of biological properties such as anthelmintic, antihistaminic, antifungal, and antibacterial [5–7]. The synthesis and characterization of new Schiff bases related to benzamides with antibacterial and antioxidant agents are of great importance for their potential pharmacological use.

General investigation revealed that the components with antimicrobial activity have gained increasing importance due to growing worldwide concern about the increase in the rate of infection by pathogenic microbes [8]. This has rendered the current available antimicrobial agents insufficient to control microbial infections and created major public health problem [9, 10]. In addition, it is known that antifungal drugs do not have selective activity because of the biochemical similarity between human cell and fungi forms. Therefore, there are many studies focused on antibacterial and antifungal compounds [11–13].

Free radicals and oxygen derivatives are constantly generated *in vitro* by a specific metabolic process [14]. These radicals can easily react with most biological molecules including proteins, lipids, lipoproteins, and DNA. These can be responsible for wide range of human conditions, such as arthritis, haemorrhagic shock, coronary artery diseases, cataract, cancer, AIDS, and age-related degenerative brain diseases [15]. Hence, there is a constant need for searching new and effective therapeutic agents.

Proteins are the most abundant macromolecules in cells and are crucial to maintaining normal cell functions. Bovine serum albumin (BSA), one of the major components in plasma protein, plays an important role in transporting and metabolizing of many endogenous and exogenous compounds in metabolism [16]. In this work, BSA was chosen as a target protein molecule for studying the interaction because of its medically important, unusual ligand-binding properties, availability, and structural homology with human serum albumin (HSA) [17].

Based on these findings, it was of interest to synthesize a new series of biologically active Schiff bases related to substituted benzamides and evaluate their antimicrobial studies by disc diffusion method and antioxidant properties by DPPH free radical scavenging and superoxide radical scavenging, with the hope to obtain more active and less toxic synthetic antimicrobial and antioxidant agents. In addition, the interaction between the NABP and BSA has been investigated using fluorescence and UV-vis absorption spectroscopic methods.

## 2. Experimental

### 2.1. Materials and Methods

All the chemicals and solvents were of AR grade. Solvents were used as supplied by commercial sources without any further purification. BSA (essentially fatty-acid-free) was purchased from Sigma Aldrich Bangalore and stored in refrigerator at 4.0°C. BSA solution was prepared in the Tris-HCl buffer solution (0.05 mol L^−1^ Tris, 0.15 mol L^−1^ NaCl, pH 7.4) and it was kept in the dark at 298 K. The compounds were prepared as stock solutions using DMF. All other reagents were of analytical reagent grade, and double-distilled water was used during the experiment.

### 2.2. Optical Measurements

Elemental analysis (C, H, N) was determined using a Carlo-Erba 1160 elemental analyzer. IR spectra were recorded on a JASCO FTIR-8400 spectrophotometer using Nujol mulls. The ^1^H-NMR and ^13^C NMR spectra were recorded on a Varian AC 400 spectrometer instrument in the indicated solvent using TMS as the internal standard. Low-resolution ESI-MS spectra were obtained on a Varian 1200L model mass spectrometer (solvent: CH_3_OH). Melting points were determined with a Buchi 530 melting point apparatus in open capillaries and are uncorrected. Compound purity was checked by thin layer chromatographic method (TLC) on precoated silica gel plates (Merck, Kieselgel 60 F254, layer thickness 0.25 mm). The fluorescence measurements were performed on a fluorophotometer (Varioskan Flash 4.00.53) and the UV-vis absorption spectra were recorded with a UV-vis spectrophotometer (Systronics 118, India). 

### 2.3. Synthesis of N-(4-((benzofuran-2-ylmethylene)amino)phenyl)acetamide (Schiff Base) ** (3)**

Schiff base was synthesized by the condensation of p-aminoacetanilide with 2-benzofurancarboxaldehyde in 1 : 1 ratio. To a solution of p-aminoacetanilide (10 mmol 1.50 g) in 20 mL ethanol required aldehyde, that is, benzofurancarboxaldehyde (10 mmol 1.46 g) was added and the reaction mixture was then stirred and refluxed over night. The solvent was evaporated under reduced pressure to obtain 2.34 g (84%) of yellow solid. ^1^H NMR (CDCl_3_, *δ* ppm): 9.07 (s, 1H, NH), 8.47 (s, 1H, –CH=N–), 7.25–7.67 (m, 9H, Ar-H), 2.20 (t, 3H, CH_3_). IR (nujol, cm^−1^): 1663 (C=O), 1603 (C=N), 1186 (NH). MS, m/z: 279 (M+1). Anal. calcd. for (C_17_H_14_N_2_O_2_): C, 73.37; H, 5.07; N, 10.07. found: C, 73.30; H, 5.01; N, 10.02.

### 2.4. Synthesis of N-acetyl-N-(4-((benzofuran-2-ylmethylene)amino)phenyl)derivatives ****(5a–j)****

Compounds **5a**–**j** were prepared by the reaction of compound **3** (0.01 mol, 0.279 g) with corresponding substituted acid chlorides (**4a**–**f**) (0.01 mol), in CH_2_Cl_2_, and the reaction mixture was stirred at room temperature for 20 h and the progress of the reaction was monitored on TLC. The reaction mixture was diluted with CH_2_Cl_2_ and washed with water (50 mL) and brine solution (100 mL), and the combined organic portions were dried over anhydrous Na_2_SO_4_. The crude product was obtained after evaporation of the solvent and recrystallization from absolute ethanol gave desired compounds **5a**–**j**.

#### 2.4.1. N-acetyl-N-(4-((benzofuran-2-ylmethylene)amino)phenyl)-2,5-difluorobenzamide ****(5a)****

^1^H NMR (CDCl_3_, *δ* ppm): 8.47 (s, 1H, –CH=N–), 7.26–7.67 (m, 12H, Ar-H), 2.20 (t, 3H, CH_3_). MS, m/z: 419 (M+1). ^13^C NMR (DMSO-d_6_, 400 MHz), *δ* (ppm): 27.4, 105.1, 112.7, 114.9, 118.6, 120.3, 121.3, 122.7, 122.9, 123.8, 125.3, 126.1, 126.7, 130.1, 130.4, 134.6, 135.9, 144.1, 145.6, 154.9, 158.3, 160.2, 174.1, 174.3. IR (nujol, cm^−1^): 1663 (C=O), 1583 (C=N). Anal. calcd. for (C_24_H_16_F_2_N_2_O_3_): C, 68.90; H, 3.85; N, 6.70. found: C, 68.83; H, 3.81; N, 6.65. 

#### 2.4.2. N-acetyl-N-(4-((benzofuran-2-ylmethylene)amino)phenyl)-2-phenylacetamide ****(5b)****

^1^H NMR (CDCl_3_, *δ* ppm): 8.41 (s, 1H, –CH=N–), 7.32–7.89 (m, 14H, Ar-H), 3.92 (s, 2H CH_2_), 2.21 (t, 3H, CH_3_). ^13^C NMR (DMSO-d_6_, 400 MHz), *δ* (ppm): 27.4, 39.6, 105.1, 112.7, 121.4, 122.5, 122.7, 123.8, 125.3, 126.7, 128.1, 129.4, 129.8, 130.2, 130.4, 130.5, 131.3, 132.5, 134.6, 135.6, 144.1, 145.6, 155.7, 167.3, 174.1. IR (nujol, cm^−1^): 1650 (C=O), 1596 (C=N). MS, m/z: 397 (M+1). Anal. calcd. for (C_25_H_20_N_2_O_3_): C, 75.74; H, 5.08; N, 7.07. found: C, 75.63; H, 5.02; N, 7.01.

#### 2.4.3. N-acetyl-N -(4-((benzofuran-2-ylmethylene)amino)phenyl)benzamide ****(5c)****

^1^H NMR (CDCl_3_, *δ* ppm): 8.37 (s, 1H, –CH=N–), 7.28–7.94 (m, 14H, Ar-H), 2.20 (t, 3H, CH_3_). ^13^C NMR (DMSO-d_6_, 400 MHz), *δ* (ppm): 27.4, 106.1, 112.5, 121.4, 122.7, 122.9, 123.8, 125.3, 126.1, 128.3, 128.5, 129.1, 129.3, 130.2, 130.4, 133.1, 134.5, 134.7, 135.9, 144.1, 145.1, 158.3, 174.1, 174.3. IR (nujol, cm^−1^): 1658 (C=O), 1603 (C=N). MS, m/z: 383 (M+1). Anal. calcd. for (C_24_H_18_N_2_O_3_): C, 75.38; H, 4.74; N, 7.33. found: C, 75.32; H, 4.69; N, 7.30.

#### 2.4.4. 2-chlorobenzylacetyl(4-((benzofuran-2-ylmethylene)amino)phenyl)carbamate ****(5d)****

^1^H NMR (CDCl_3_, *δ* ppm): 8.41 (s, 1H, –CH=N–), 7.36–7.86 (m, 13H, Ar-H), 4.97 (s, 2H, CH_2_) 2.21 (t, 3H, CH_3_). ^13^C NMR (DMSO-d_6_, 400 MHz), *δ* (ppm): 27.4, 62.5, 105.3, 112.5, 121.3, 122.9, 123.1, 123.8, 125.4, 126.3, 127.5, 128.9, 129.7, 129.9, 130.4, 130.6, 131.7, 134.8, 135.4, 140.2, 144.1, 145.6, 155.3, 152.7, 174.2. IR (nujol, cm^−1^): 1668 (C=O), 1584 (C=N). MS, m/z: 447 (M+1). Anal. calcd. for (C_25_H_19_ClN_2_O_4_): C, 67.19; H, 4.29; N, 6.27. found: C, 67.12; H, 4.18; N, 6.20. 

#### 2.4.5. N-acetyl-N-(4-((benzofuran-2-ylmethylene)amino)phenyl)-4-nitrobenzamide ****(5e)****

^1^H NMR (CDCl_3_, *δ* ppm): 8.36 (s, 1H, –CH=N–), 7.44–8.07 (m, 13H, Ar-H), 2.24 (t, 3H, CH_3_). ^13^C NMR (DMSO-d_6_, 400 MHz), *δ* (ppm): 27.4, 105.1, 112.7, 121.3, 122.7, 122.9, 123.8, 124.5, 124.7, 125.3, 126.1, 130.1, 130.3, 130.8, 131.0, 134.8, 135.4, 141.3, 144.1, 145.3, 151.3, 158.3, 174.1, 174.3. IR (nujol, cm^−1^): 1650 (C=O), 1603 (C=N). MS, m/z: 428 (M+1). Anal. calcd. for (C_24_H_17_N_3_O_5_): C, 67.44; H, 4.01; N, 9.83. found: C, 67.42; H, 3.98; N, 9.76.

#### 2.4.6. N-acetyl-N-(4-((benzofuran-2-ylmethylene)amino)phenyl)-2-methylbenzamide ****(5f)****

^1^H NMR (CDCl_3_, *δ* ppm): 8.39 (s, 1H, –CH=N–), 7.28–7.84 (m, 13H, Ar-H), 4.01 (s, 2H, CH_2_), 2.20 (t, 3H, CH_3_), 1.18 (s, 3H, CH_3_). ^13^C NMR (DMSO-d_6_, 400 MHz), *δ* (ppm): 19.6, 27.4, 105.3, 112.5, 121.4, 122.8, 123.0, 124.1, 125.7, 126.0, 128.1, 129.2, 130.5, 130.7, 131.8, 133.0, 134.5, 135.1, 136.4, 138.2, 144.4, 145.9, 158.1, 174.1, 174.3. IR (nujol, cm^−1^): 1660 (C=O), 1601 (C=N). MS, 397 (M+1). Anal. calcd. for (C_25_H_20_N_2_O_3_): C, 75.74; H, 5.08; N, 7.07. Found: C, 75.69; H, 5.01; N, 7.04. 

#### 2.4.7. N-acetyl-N-(4-(benzofuran-2-ylmethyleneamino)phenyl)-3,4,5-trifluorobenzamide ****(5g)****

^1^H NMR (CDCl_3_, *δ* ppm): 8.39 (s, 1H, –CH=N–), 7.29–7.63 (m, 11H, Ar-H), 2.21 (t, 3H, CH_3_). ^13^C NMR (DMSO-d_6_, 400 MHz), *δ* (ppm): 27.3, 105.2, 110.4, 110.7, 112.9, 121.7, 122.7, 122.9, 124.3, 124.9, 125.4, 130.2, 130.4, 133.4, 134.6, 135.2, 144.1, 144.3, 145.6, 158.1, 160.7, 160.9, 174.1, 174.3. IR (nujol, cm^−1^): 1655 (C=O), 1601 (C=N). MS, m/z: 437 (M+1). Anal. calcd. for (C_24_H_15_F_3_N_2_O_3_): C, 66.06; H, 3.46; N, 6.42. Found: C, 66.01; H, 3.42; N, 6.40. 

#### 2.4.8. N-acetyl-N-(4-((benzofuran-2-ylmethylene)amino)phenyl)-3,5-difluorobenzamide ****(5h)****

^1^H NMR (CDCl_3_, *δ* ppm): 8.49 (s, 1H, –CH=N–), 7.45–7.73 (m, 12H, Ar-H), 2.20 (t, 3H, CH_3_). ^13^C NMR (DMSO-d_6_, 400 MHz), *δ* (ppm): 27.4, 105.1, 109.3, 110.4, 110.6, 112.7, 121.3, 122.9, 123.1, 124.1, 125.37, 126.0, 130.6, 130.8, 134.6, 135.4, 138.7, 144.2, 145.3, 158.3, 162.0, 162.3, 174.2, 174.5. IR (nujol, cm^−1^): 1662 (C=O), 1597 (C=N). MS, m/z: 419 (M+1). Anal. calcd. for (C_24_H_16_F_2_N_2_O_3_): C, 68.90; H, 3.85; N, 6.70. found: C, 68.81; H, 3.80; N, 6.65. 

#### 2.4.9. N-acetyl-N-(4-(benzofuran-2-ylmethyleneamino)phenyl)-4-methoxybenzamide ****(5i)****

^1^H NMR (CDCl_3_, *δ* ppm):, 8.81 (s, 1H, –CH=N–), 7.51–7.76 (m, 13H, Ar-H), 3.86 (s, 3H, OCH_3_), 2.25 (t, 3H, CH_3_). ^13^C NMR (DMSO-d_6_, 400 MHz), *δ* (ppm): 27.3, 57.9, 105.3, 112.5, 115.1, 115.3, 121.3, 122.7, 122.9, 124.3, 124.9, 125.4, 127.7, 129.3, 129.5, 130.1, 130.3, 134.6, 135.2, 142.4, 146.9, 158.3, 167.0, 174.1, 174.3. IR (nujol, cm^−1^): 1658 (C=O), 1600 (C=N). MS, m/z: 413 (M+1). Anal. calcd. for (C_25_H_20_N_2_O_4_): C, 72.80; H, 4.89; N, 6.79. Found: C, 72.69; H, 4.80; N, 6.72. 

#### 2.4.10. N-acetyl-N-(4-((benzofuran-2-ylmethylene)amino)phenyl)-4-hydroxybenzamide ****(5j)****

^1^H NMR (CDCl_3_, *δ* ppm): 10.02 (s, 1H, OH), 8.32 (s, 1H, –CH=N–), 7.56–8.07 (m, 13H, Ar-H), 2.23 (t, 3H, CH_3_). ^13^C NMR (DMSO-d_6_, 400 MHz), *δ* (ppm): 27.3, 105.7, 112.1, 117.4, 117.6, 121.9, 122.7, 122.9, 124.3, 125.1, 125.4, 127.3, 129.4, 129.6, 130.1, 130.3, 134.6, 135.3, 144.4, 146.2, 158.7, 163.9, 174.7, 174.9. IR (nujol, cm^−1^): 3434 (OH), 1664 (C=O), 1603 (C=N). MS, m/z: 399 (M+1). Anal. calcd. for (C_24_H_18_N_2_O_4_): C, 72.35; H, 4.55; N, 7.03. found: C, 72.30; H, 4.52; N, 7.01.

### 2.5. Antimicrobial Activity

#### 2.5.1. Microbial Strains

The *in vitro* antimicrobial screening effects of the compounds (**3** and **5a–j**) were individually tested against a panel of bacteria and fungi including *Staphylococcus aureus* (NCIM 5021), *Bacillus subtilis* (NCIM 2999), *Pseudomonas aeruginosa* (NCIM 5029), *Escherichia coli* (NCIM 2574), *Candida. albicans* (NCIM 3471), and *Aspergillus flavus* (NCIM 524). Microbial strains were cultured overnight at 37°C in nutrient and potato dextrose agar medium. All the pure microbial strains were obtained from National Chemical Laboratory (NCL), Pune, India.

#### 2.5.2. Antimicrobial Screening

The antibacterial activity of compounds was determined by agar disc diffusion method [18]. Briefly, a suspension of tested bacterial strains was spread on the nutrient agar medium and potato dextrose agar for fungi. The discs (6 mm in diameter) impregnated with test chemicals each dissolved in concentration (100 *μ*g/mL in DMSO) were placed on the inoculated agar and these plates were kept at 4°C for 2 h. The plate was incubated at 37°C for 24 h in case of bacteria and 48 h at 28°C in case of fungi. streptomycin (10 *μ*g/disc) and fluconazole (10 *μ*g/disc) were used as the standards for antibacterial and antifungal activity, respectively. All the samples were performed in triplicate and the zone of inhibition was measured in millimeters.

#### 2.5.3. Minimum Inhibitory Concentration (MIC)

The minimum inhibitory concentration of the synthesized compounds was determined by dilution method [19]. The compounds were dissolved and then diluted using DMSO; two-fold serial concentrations of the compounds were employed to determine the MIC. In this method, the test concentrations of chemically synthesized compounds were made from 5 to 125 *μ*g/mL. The MIC value was determined as the lowest concentration of the compound that completely inhibited macroscopic growth of microorganism.

### 2.6. Antioxidant Activity

#### 2.6.1. DPPH Free Radical Scavenging Activity

The capacity to scavenge the stable free radical 2,2-diphenyl-1-picrylhydrazyl (DPPH) was monitored according to the Blois method [20]. The test samples (10–100 *μ*L) were mixed with 1 mL of DPPH (0.1 mM) solution and filled up with methanol to a final volume of 4 mL. Absorbance of the resulting solution was measured at 517 nm in a visible spectrophotometer (Model 166, Systronics, India). The free radical scavenging rate of the reaction solution was calculated as a percentage (%) of DPPH decolouration using the equation

(1) $$\begin{array}{c} \text{I}\left(\%\right)=\left(\frac{A_{\text{blank}}-A_{\text{sample}}}{A_{\text{blank}}}\right)\times 100, \end{array}$$

where *A*_blank_ is the absorbance of the control reaction mixture excluding the test compounds, and *A*_sample_ is the absorbance of the test compounds. Radical scavenging potential was expressed as IC_50_ value, which represents the sample concentration at which 50% of the DPPH radicals were scavenged. Tests were carried out in triplicate and the results were expressed as mean values ± standard deviations.

#### 2.6.2. Superoxide Radical Scavenging Assay

Superoxide radical scavenging activity was measured as described by Kovala-Demertzi et al., (2004) [21]. The assay is based on the reduction of nitroblue tetrazolium (NBT) by superoxide ions generated by the xanthine-xanthine oxidase system (X–XO). The reaction system contained 0.2 mM xanthine and 0.6 mM NBT in 0.1 M phosphate buffer, pH 7.8. The tested compounds were dissolved in methanol. The reaction was started by addition of XO (0.07 U mL^−1^), an activity which allowed to yield the absorbance change between 0.03 and 0.04 per minute, at 560 nm. The extent of NBT reduction was followed spectrophotometrically, by measuring the increase of the absorbance at 560 nm. All the experiments were replicated three times. The IC_50_ of each compound was defined as the concentration which inhibited 50% of the NBT reduction by O_2_^•−^ produced in the X–XO system.

### 2.7. Measurement of Binding Parameters

For each of the four active compounds (**5a**, **5c**, **5g**, **5h**, **5i**, and **5j**), the binding parameters with BSA molecules were measured by fluorescence spectroscopy. A solution (2.5 mL) containing 1.00 × 10^−5^ mol L^−1^ BSA was titrated by successive additions of 1.00 × 10^−3^ mol L^−1^ NABP stock solution and the concentration of NABP varied from 0 to 3.0 × 10^−5^ mol L^−1^ (a–g). Titrations were done manually by using microinjector. Fluorescence intensity was measured (excitation at 280 nm and emission at 340 nm). All experiments were performed at 298 K.

## 3. Results and Discussion

### 3.1. Chemistry

**Scheme 1:**
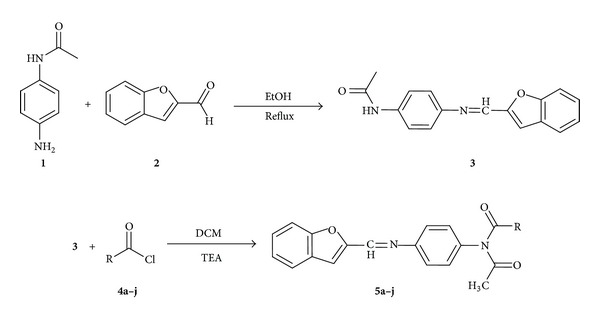
Synthetic route of amide derivatives **5a–j**.

The synthetic route for the preparation of benzamide compounds is outlined in Scheme 1. Synthesis of the desired compound **3** was achieved by condensation of *p*-aminoacetanilide **1** with 2-benzofurancaroxaldehyde **2** according to the known method [22]. The second synthetic step involves the amidation of compound **3** with different substituted acid chlorides (**4a–j**) in presence of triethylamine in dichloromethane giving rise to the respective aryl amide derivatives (**5a–j**). The compounds were purified by repeated recrystallization from ethanol and then dried. The physical and chemical properties of all the synthesized compounds are given in Table 1. The elemental analyses, ^1^H NMR, ^13^C NMR, and FTIR spectra are fully consistent with the structure and the melting points are sharp, indicating the purity of the prepared Schiff bases. 

In the ^1^H NMR spectra, the chemical shift and multiplicity patterns correlated well with the proposed structures. The formation of compound **3** was supported by the ^1^H NMR spectrum (see Supplementary Material available online at http://dx.doi.org/10.1155/2013/791591) by the appearance of a sharp singlet at 8.47 ppm, corresponding to the azomethine (–CH=N–) proton. The multisignals within the range 7.25–8.07 ppm are assigned to the aromatic protons. ^1^H NMR spectra of all synthesized compounds (**5a–j**) showed–CH=N– proton as singlet at 8.32–8.81 ppm. A comparison of ^1^H NMR spectra of compounds **5a–j** with the **3** has shown disappearance of signal of NH (9.07 ppm) proton on the formation of amide bond. The signal due to –OH in the compound **5j** appeared as singlet at 10.02 ppm. The aromatic protons were observed at expected regions. ^1^H NMR spectra of all compounds showed singlet at 2.20–2.25 ppm due to N-acetyl group (N–CO–CH_3_).

The absence of NH_2_ and C=O absorption bands in the IR spectra confirmed that the synthesized compounds were obtained *via* condensation. The IR spectrum showed a characteristic strong absorption band at 1603 cm^−1^ which is attributed to the stretching vibration of C=N bond formation in the synthesized compound **3**. The IR spectra of **5a–j** revealed the presence of C=O stretching band at 1650–1668 cm^−1^ in all the analogues and **5j** showed broad phenolic stretching at 3434 cm^−1^. Mass spectra of all newly synthesized compounds showed M+1 peak, in agreement with their molecular formula. 

### 3.2. Antimicrobial Activity

In testing the antibacterial and antifungal activity of these compounds, more than one test organism was used to increase the chance of detecting antibiotic principles in tested materials. The antimicrobial activity of the newly synthesized compounds (**5a–j**) was evaluated against two gram-positive bacteria (*B. subtilis* and *S. aureus*), two gram-negative bacteria (*E. coli* and *P. aeruginosa*), and two fungi (*A. niger* and *A. flavus*). Minimum inhibitory concentration (MIC) of these compounds against bacteria and fungi was determined. Standard antibiotics, namely, streptomycin and standard antifungal drug fluconazole were used for comparison with antibacterial and antifungal activities shown by compounds (Table 2). All the compounds possessed moderate to good antibacterial activity.

The obtained compounds display good antibacterial activity. In case of antibacterial activity, all the compounds possess higher antibacterial activity than the compound **3**. The most potent antibacterial activity exhibited by compound **5g** (MIC 9.8 *μ*g/mL) with three fluoro groups substituted ring in the respective series compared to the compounds bearing other electron-donating or -withdrawing groups. The compounds **5a** and **5h** possess active inhibition against all the tested strains; this may be due to the fact that the presence of two fluoro groups in the compound is responsible for the enhancement of activity. Other compounds exhibit moderate to good antibacterial activity against all organisms. Among these compounds, **5b** and **5f** exhibit poor antibacterial activity against *S. aureus*, *E. coli*, and *P. aeruginosa* as compared to the standard drug.

The screening data of antifungal activity of these series of compounds shows wide range of antifungal activity. Similarly, compounds **5a**, **5e**, **5g**, and **5h** exhibit good antifungal activity against *Candida albicans* and *Aspergillus flavus* compared to the standard drug fluconazole. In the whole series, compound **5g** with MIC 10 *μ*g/mL shows the highest percentage of inhibition against both fungal strains and the compounds **5a** and **5h** pose difference in activity due to presence of fluoro group at different positions. It is interesting to note that the electron-withdrawing property of the phenyl ring is important, which is corroborated by eminent activity of compounds with halogen group and decreased activity of compounds with either hydroxyl, methyl, or methoxy group in the phenyl ring; hence the replacement of electron-donating group in place of halogens shows decrease in antimicrobial activity. The researches of structure-activity relationship show that halogen atoms on phenyl ring enhanced the biological activity effectively [23]. It is because halogen could improve the affinity between compounds and special target of bacteria.

### 3.3. Antioxidant Activity

DPPH radical scavenging activity evaluation is a standard assay in antioxidant activity studies and offers a rapid technique for screening the radical scavenging activity of specific compounds [24]. The free radical scavenging activity of newly synthesized compounds was tested by their ability to bleach the stable radical DPPH. The activity was monitored by following the absorption at 517 nm in a visible spectrophotometer. In the presence of any free radical scavenger, this odd electron pairs up and causes the diminishing of absorption band which is proportional to the number of electrons taken up. The activity was studied at different concentrations for each compound. A freshly prepared DPPH solution exhibits a deep purple color with an absorption maximum at 517 nm. Resulting from a color change from purple to yellow the absorbance decreased when the antioxidant molecule can quench DPPH free radical through donation of hydrogen atom or by electron donating to form a stable DPPH molecule [25]. Hence, more rapidly the absorbance decreases, the more potent the antioxidant activity of the compound becomes.

The scavenging effect of the synthesized compounds was reported in Table 3. All the compounds (**5a–j**) showed comparable or slightly less activity to the standard ascorbic acid. The antioxidant activity was expressed as the 50% inhibitory concentration (IC_50_) based on the amount of compound required for a 50% decrease of the initial DPPH radical concentration. It was observed that the entire compounds notably reduced the concentration of DPPH free radical. It is clearly demonstrated that free radical scavenging increases with increasing concentration.

The better activity of compound **5j** (IC_50_ 15.3 ± 0.11 *μ*g/mL), having hydroxyl group at *p*-position in the aromatic ring, has high electron-releasing properties and it activates aromatic ring. The compound **5i** (IC_50_ 18.5 ± 0.17 *μ*g/mL), bearing an electron-donating methoxy group at para position, showed the better DPPH radical scavenging activity compared to electron-withdrawing group substituted to phenyl ring. The compounds **5g** and **5h** (IC_50_ > 100 *μ*g/mL) exhibit less activity than that of standard and **5j**, due to the presence of electron-withdrawing group. Observing the overall data for antioxidant activity, it is clear that compounds **5i** and **5j** were found to be the most effective antioxidants among all compounds.

Superoxide anion radical is normally initially formed, and its effects can be magnified because it produces other kinds of free radicals and oxidizing agents [26]. The enzymatic superoxide anion radical was generated by the xanthine/xanthine oxidase reaction system. The production of superoxide was estimated by the nitroblue tetrazolium method [27]. The results (Table 3) revealed that all of the tested compounds exhibited superoxide activity with IC_50_ in the range of 11.6–123 *μ*g/mL. It should be noted that the activity of the compounds **5i** and **5j** was comparable to that of BHA as observed from the IC_50_ values. Compounds **5i** and **5j** showed very good activity, since these compounds are having methoxy and hydroxyl groups at *p*-position in the ring, respectively. Differently, in the *m*-position, methoxy and hydroxyl groups act as an electron-withdrawing properties, (positive mesomeric effect is higher than negative inductive effect), which are possible for providing a destabilizing effect [28, 29].

### 3.4. Fluorescence Quenching of BSA by NABP

Fluorescence spectroscopy is a powerful tool for the study of the reactivity of chemical and biological systems since it allows nonintrusive measurements of substances in low concentration under physiological conditions [30]. Fluorescence quenching refers to any process which decreases the fluorescence intensity of a sample induced by a variety of molecular interactions with quencher molecule, including excited-state reactions, molecular rearrangements, energy transfer, ground-state complex formation, and collisional quenching processes. 

**Figure 1:**
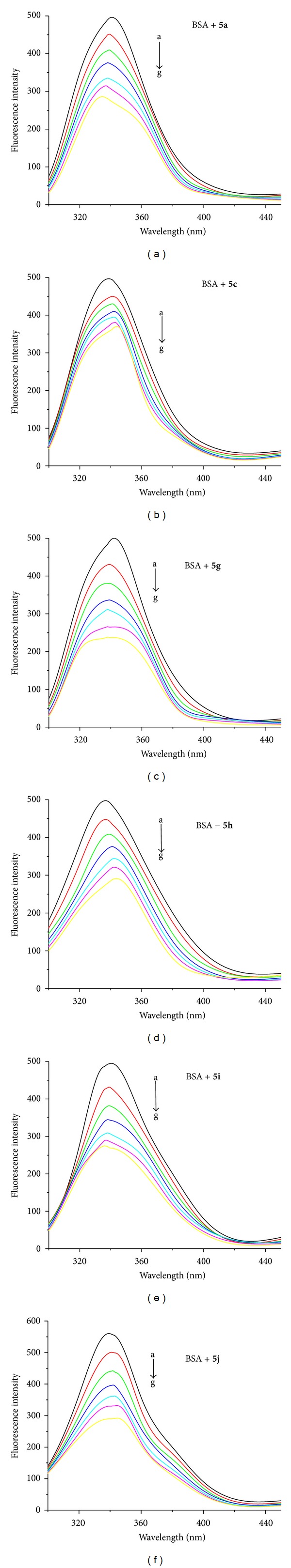
Fluorescence quenching spectra of BSA by NABP (**5a**,** 5c**,** 5g**,** 5h**,** 5i**,** 5j**) at 298 K. c(BSA) = 1.00 × 10^−5^ mol L^−1^; c(NABP): 0, 0.5, 1.0, 1.5, 2.0, 2.5, 3.0 (×10^−5^ mol L^−1^) from a to g, respectively.

The fluorescence spectra of BSA in the presence of different concentrations of NABP are shown in Figure 1. It is well known that BSA emits a strong fluorescence peaked at around 340 nm with excitation wavelength of 280 nm. Viewed from Figure 1, the presence of NABP led the emission intensity of BSA to decrease regularly, which indicated that the NABP can interact with BSA and quenches its intrinsic fluorescence. A remarkable red shift of BSA fluorescence emission maximum wavelength is observed in **5c**, **5j**, and **5h**. Red shift observed upon interaction of BSA with the compounds **5c**, **5j**, and **5h** indicates that change in conformation of tryptophan environment in BSA. The polarity around the tryptophan residue increases whereas its hydrophobicity decreases [31]. Furthermore, a slight blue shift of the maximum emission wavelength was observed for **5a**, **5g**, and **5i**. The phenomena imply that the polarity around the tryptophan residue decreases with the increasing hydrophobicity after addition of **5a**, **5g**, and **5i** to BSA. 

### 3.5. Fluorescence Quenching Mechanism: Identification of Binding Site and Binding Constant

In order to investigate the quenching mechanism, the fluorescence quenching data *K*_0_ and *n* were analyzed by the Stern-Volmer equation (2) [32]:

(2) $$\begin{array}{c} \frac{F_{0}}{F}=1+K_{\text{sv}}\left[Q\right]=1+K_{q}\tau_{0}\left[Q\right], \end{array}$$

where *F*_0_ and *F* denote the steady-state fluorescence intensities in the absence and in the presence of quencher, respectively, *K*_0_ is binding constant of BSA, [*Q*] is the equilibrium concentration of the quencher, and *n* is the number of binding sites. The average lifetime, *τ*_0_ of the molecule without quencher is 10^−8^ s [33].

**Figure 2:**
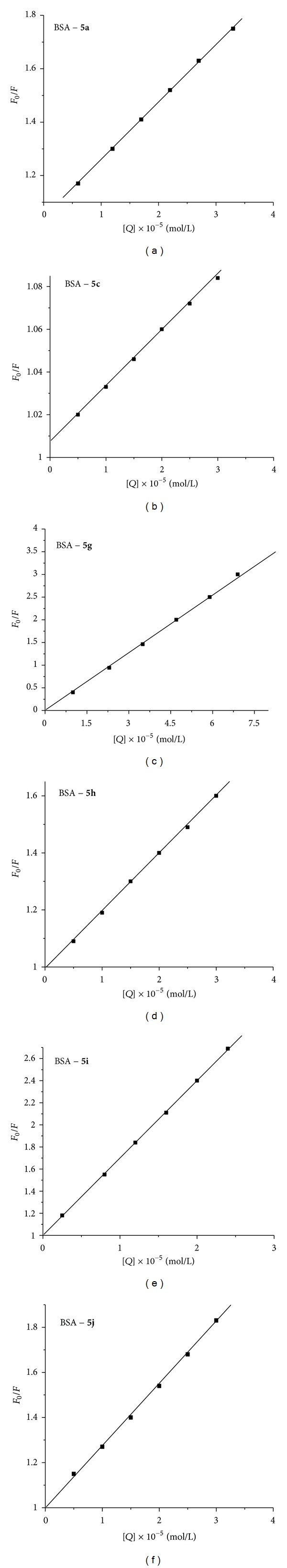
Van't Hoff's plots for the interaction of BSA and NABP.

The Stern-Volmer plots for the interaction of compound with BSA at temperature (*T* = 298 K) are shown in Figure 2. In all cases the plots are fairly linear. From Figure 2, the values of *K*_sv_ and *K*_*q*_ were obtained and listed in Table 4. Dynamic and static quenching can be distinguished by the quenching constant *K*_*q*_ [34]. The observed values of *K*_*q*_ were larger than the maximum scattering collision quenching constant 2.0 × 10^10^ L mol^−1^ s^−1^, which suggests that the fluorescence quenching mechanism between NABP and BSA may be static rather than dynamic [35]. 

When the quenching mechanism is static, the binding parameters between BSA and compounds can be determined using (3):

(3) $$\begin{array}{c} \log\frac{\left(F_{0}-F\right)}{F}=\log\;K_{\text{a}}+n\;\log\left[Q\right]. \end{array}$$

The plot of log⁡[(*F*_0_ − *F*)/*F*] versus log⁡[NABP], binding constant *K*_a_, and binding sites *n* were calculated from the intercept and slope. Figure 2 shows the log⁡[(*F*_0_ − *F*)/*F*] versus log⁡[NABP] and Table 5 gives the corresponding calculated results. The value of n approximately equaled 1. 

The substitutions on the benzene ring could enhance the binding affinity of BSA and NABP. As shown in Table 5, the binding constants of the interaction between them increased in the following order: **5j**  <  **5c**  <  **5h**  <  **5g**  <  **5a**  <  **5i**. The two aspects that may result in binding potency are as follows: (1) the process of NABP-BSA binding is promoted strongly by the polarity of the substituent; (2) the molecular size of NABP, the large-size molecule, may have larger hydrophobic area which can interact with hydrophobic surface on the protein molecule. In this work, the molecular size and polarity play a significant role in the binding between NABP and BSA.

### 3.6. Energy Transfer from BSA to Compound

**Figure 3:**
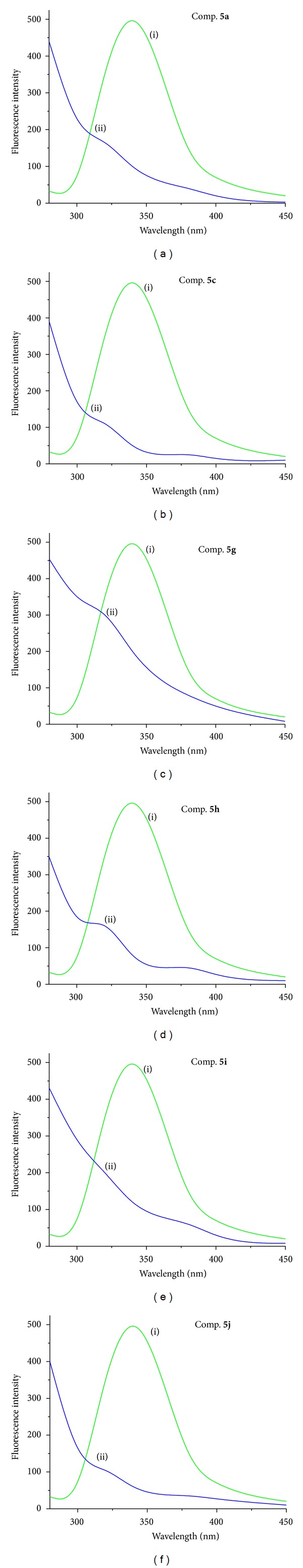
The overlap of the fluorescence spectrum of BSA (i) and the absorbance spectrum of NABP (ii).

The overlap of the UV absorption spectra of NABP with the fluorescence emission spectra of BSA is shown in Figure 3. 

The binding distance (*r*) between a protein residue (donor) and a bound drug molecule (acceptor) can be calculated from Foster's nonradiative energy transfer theory. The efficiency of energy transfer *E* and the distance between the acceptor and donor *r* can be defined as the following equation [36]:

(4) $$\begin{array}{c} E=1-\left(\frac{F}{F_{0}}\right)=\frac{R_{0}^{6}}{R_{0}^{6}}+r^{6}, \end{array}$$

where *r* is the distance between the acceptor and donor; *F* and *F*_0_ are the fluorescence intensities of BSA in the presence of and in the absence of quencher, respectively; *R*_0_ is the critical energy transfer distance when the transfer efficiency is 50%. It is given by the following equation:

(5) $$\begin{array}{c} R_{0}^{6}=8.8\times 10^{-25}K^{2}N^{-4}\Phi J. \end{array}$$

*K* ^2^ is a factor describing the spatial orientation factor related to the geometry of the donor and acceptor of dipoles; *N* is the refractive index of medium; Φ is the fluorescence quantum yield of the donor in the absence of acceptor; *J* is the effect of spectra overlap between the fluorescence emission spectrum of the donor and the UV-vis absorption spectrum of the acceptor, and *J* can be calculated by (6):

(6) $$\begin{array}{c} J=\frac{f\left(\lambda \right)\varepsilon \left(\lambda \right)\lambda^{4}\Delta \lambda }{\Sigma F\left(\lambda \right)\Delta \lambda }, \end{array}$$

where *F*(*λ*) is the corrected fluorescence intensity of the donor in the wavelength range from *λ* to *λ* + Δ*λ* and *ε*(*λ*) is the molar absorption coefficient of the acceptor at wavelength *λ*. In the present case, *K* ^2^ = 2/3, *N* = 1.311, and Φ = 0.118 for BSA molecules [37], respectively. And then, the values of *J*, *E*, *R*_0_, and *r* were calculated and shown in Table 6. The binding distance is shorter than 8 nm which indicates that the energy could transfer from BSA to NABP with high possibility [38].

## 4. Conclusion

In this work, we have presented the synthesis of novel benzamides related to Schiff base in good yield and their spectroscopic characterization by means of different spectral studies and their antimicrobial and antioxidant activities have been evaluated. The antimicrobial evaluation data revealed that among the compounds studied, derivatives **5a**, **5e**, **5g**, and **5h** have exhibited good antibacterial activity comparable to the standard streptomycin, while compounds **5a**, **5g**, and **5h** displayed better antifungal activity comparable to the standard fluconazole. The maximum activity was observed with compounds having trifluoro substituent in the phenyl moiety. Further, the compounds **5i** and **5j** displayed pronounced antioxidant activity as interpreted by the results of DPPH and superoxide radical scavenging assays. The possible quenching mechanism of fluorescence of BSA by NABP was a static quenching by forming the BSA-NABP complexes. All the substituents can influence the binding affinity of NABP with BSA. The effect of 3,4,5-trifluoro, 4-OCH_3_, 2,5-difluoro, and 3,5-difluoro on the benzene ring enhances the binding affinity of NABP with BSA. According to Forster's theory of nonradiation energy transfer, energy transfer from BSA to NABP occurs with high probability. 

## Supplementary Material

The representative 1H NMR, IR and mass spectra of the synthesized compounds associated with this article can be given in the supplementary material.

## Figures and Tables

**Table 1:** Physical and chemical properties of compounds** 5a**–**j**.

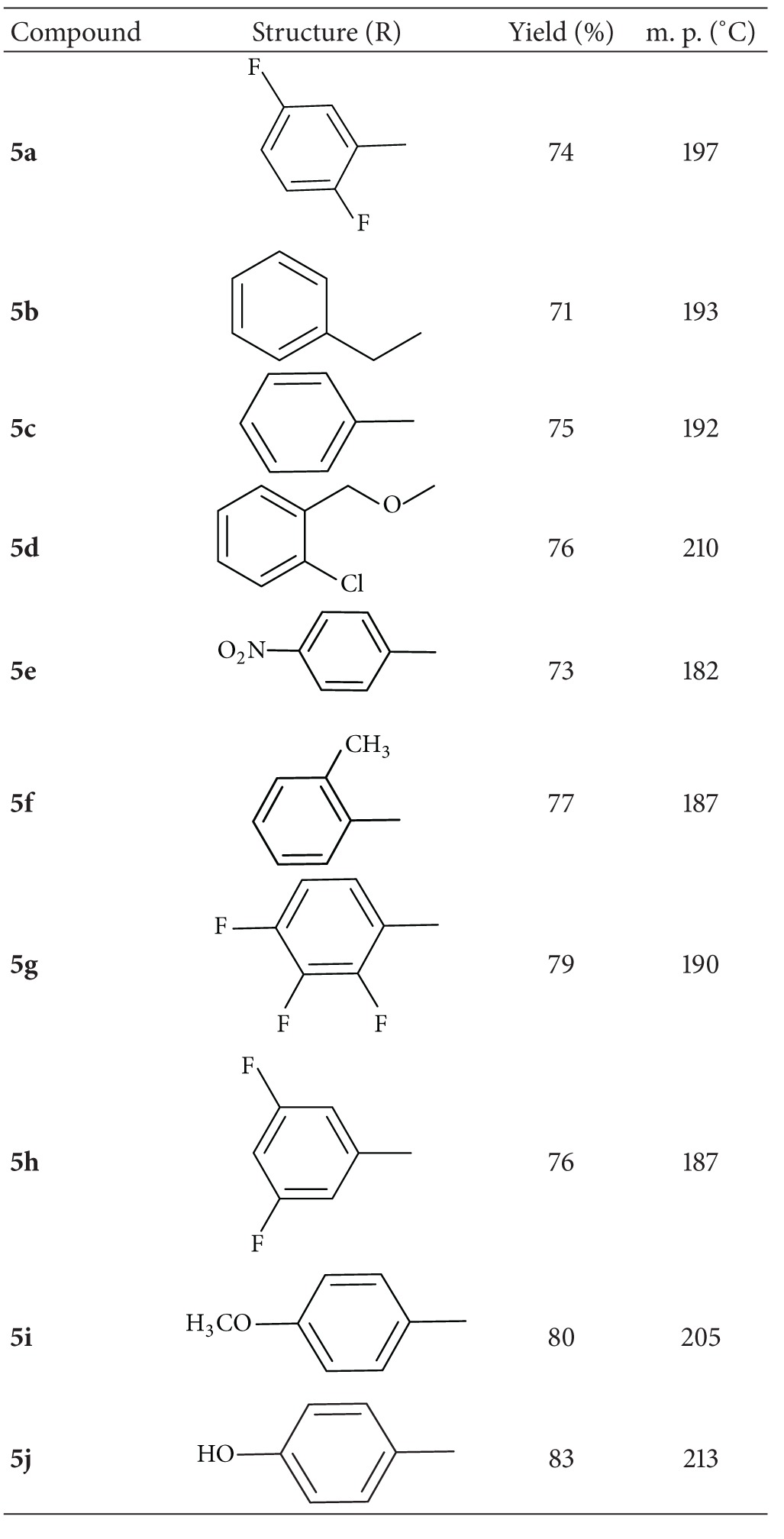

**Table 2:** Antibacterial and antifungal activity of the synthesized compounds **3** and** 5a**–**j**.

Compounds	*In vitro* activity zone of inhibition in mm (MIC in *µ*g/mL)^a^					
	Gram positive		Gram negative		Fungi	
	*S. aureus *	*B. subtilis *	*E. coli *	*P. aeruginosa *	*A. niger *	*A. flavus *
**3**	08 (75)	07 (75)	11 (>100)	10 (>100)	12.4 (75)	12.5 (75)
**5a**	11 (12)	16 (12)	12 (25)	14 (25)	11 (15)	10.4 (15)
**5b**	10 (50)	10 (30)	10 (50)	11 (30)	12 (25)	15 (25)
**5c**	11 (35)	10 (35)	15 (50)	12 (50)	09 (40)	15 (40)
**5d**	12 (30)	14 (50)	10 (50)	13 (50)	13.7 (25)	11 (40)
**5e**	14 (15)	11 (15)	09 (25)	11 (25)	14.1 (20)	13.6 (20)
**5f**	10 (50)	08 (50)	11 (100)	10 (100)	12 (100)	10.2 (75)
**5g**	13.6 (9.8)	13.5 (10)	12.7 (12)	14.0 (10)	13.2 (10)	14.0 (15)
**5h**	14 (12.4)	12 (12.4)	11 (25)	17 (25)	11.4 (10)	10.9 (15)
**5i**	14 (40)	10 (50)	11 (30)	13 (35)	10 (35)	14 (30)
**5j**	12 (35)	09 (35)	11 (50)	13 (50)	09 (40)	15 (40)
Streptomycin	14.1 (05)	13.4 (05)	16.5 (05)	14 (05)	—	—
Fluconazole	—	—	—	—	16 (05)	19 (05)

^a^Average of three replicates.

**Table 3:** DPPH radical scavenging activity of compounds** (5a–j)**.

Compound	IC_50_ (*µ*g/mL)	
	DPPH	Superoxide
**3**	95.3 ± 0.76	85.1 ± 0.33
**5a**	97.5 ± 0.37	91.3 ± 0.26
**5b**	85.3 ± 0.14	92.0 ± 0.35
**5c**	61.7 ± 0.55	60.9 ± 0.28
**5d**	48.9 ± 0.11	55.3 ± 0.04
**5e**	87.4 ± 0.31	79.2 ± 0.24
**5f**	53 ± 0.04	49 ± 0.17
**5g**	>125	123 ± 0.44
**5h**	100 ± 0.19	96.1 ± 0.31
**5i**	18.5 ± 0.17	14.3 ± 0.23
**5j**	15.3 ± 0.11	11.6 ± 0.04
AA^a^	12.6 ± 0.43	Nt^c^
BHA^b^	Nt^c^	13.4 ± 0.29

^a^Ascorbic acid, ^b^butylated hydroxyanisole.

^c^Not tested.

**Table 4:** The quenching constants of BSA by compounds.

Compound	*K*_sv_ (L mol^−1^)	*K*_*q*_ (L mol^−1^ s^−1^)	*R* ^a^
**5a**	9.96 × 10^4^	9.96 × 10^12^	0.9981
**5c**	1.93 × 10^4^	1.93 × 10^12^	0.9987
**5g**	7.98 × 10^4^	7.98 × 10^12^	0.9978
**5h**	2.13 × 10^4^	2.13 × 10^12^	0.9974
**5i**	7.40 × 10^4^	7.40 × 10^12^	0.9994
**5j**	2.39 × 10^4^	2.39 × 10^12^	0.9972

^a^Linear quotient.

**Table 5:** The binding constants and the number of binding sites of compounds with BSA.

Compound	*K*_a_ (L mol^−1^)	*n*	*R*
**5a**	5.26 × 10^5^	1.180	0.9981
**5c**	2.59 × 10^4^	0.855	0.9987
**5g**	1.57 × 10^5^	1.281	0.9998
**5h**	0.79 × 10^5^	1.136	0.9991
**5i**	6.38 × 10^5^	1.208	0.9996
**5j**	1.09 × 10^4^	0.961	0.9970

**Table 6:** The distance parameters between compounds and BSA.

Compound	*J* (cm^3^ L mol^−1^)	*R*_0_ (nm)	*E*	*r* (nm)
**5a**	1.05 × 10^−14^	2.51	0.11	3.77
**5c**	3.05 × 10^−14^	3.04	0.14	4.11
**5g**	2.37 × 10^−14^	2.86	0.31	3.07
**5h**	4.91 × 10^−14^	2.16	0.15	2.93
**5i**	2.79 × 10^−14^	2.83	0.33	3.17
**5j**	4.26 × 10^−14^	3.02	0.14	4.09
