# Supplementary material for: Synthesis, Characterization, and BSA Binding Studies of Some New Benzamides Related to Schiff Base
Source: ISRN Org Chem. 2013 Apr 7;2013:791591. doi: 10.1155/2013/791591 (PMC3767324; doi:10.1155/2013/791591)
Supplement: Supplementary file 1 — The representative 1H NMR, IR and mass spectra of the synthesized compounds associated with this article can be given in the supplementary material. [file 791591.f1.doc]

**Synthesis, Characterization and BSA Binding Studies of Some New Benzamides Related to Schiff base**

**Supplementary file:**

**
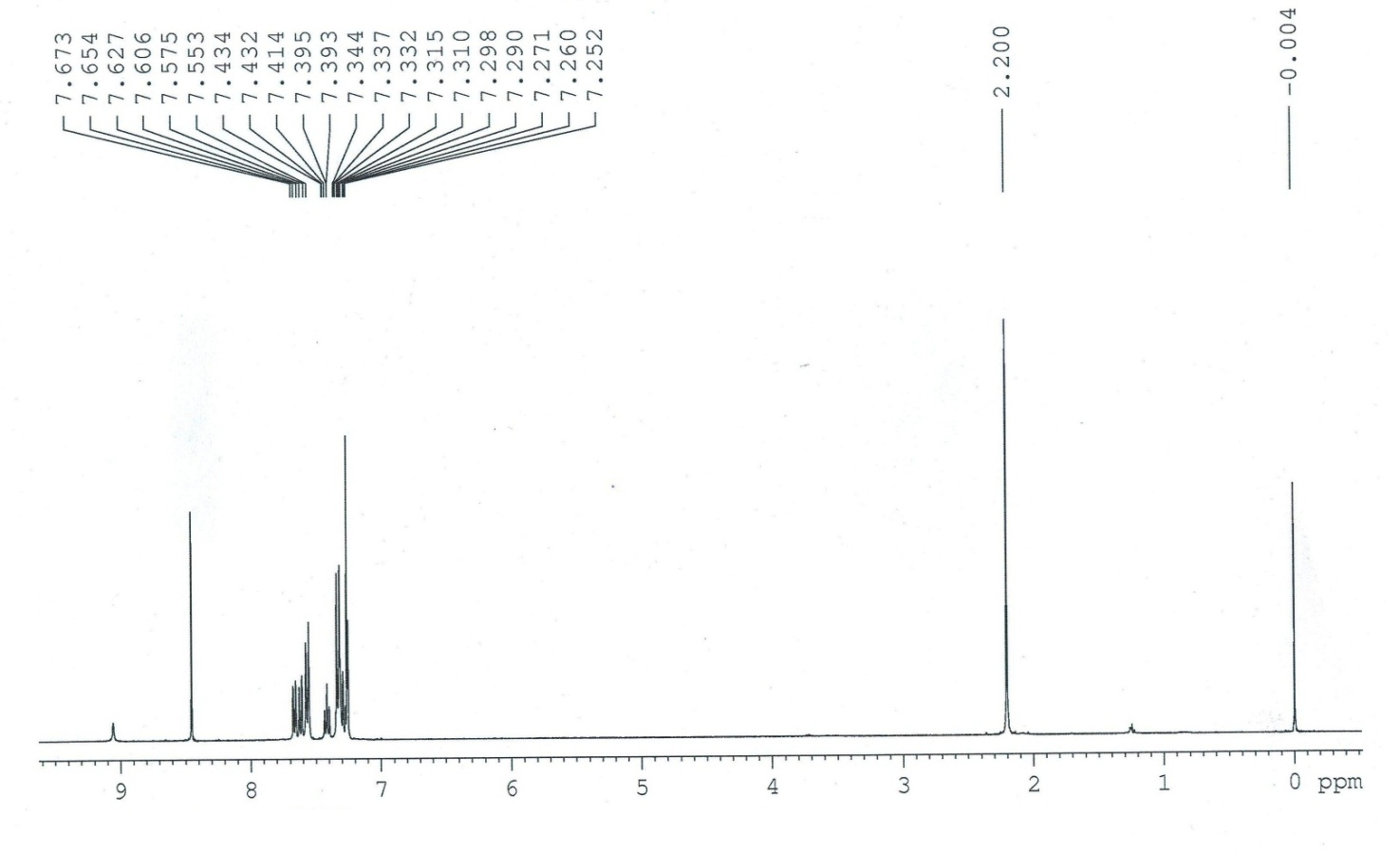
**

**S1:** ^1^H NMR spectrum of *N*-(4-((benzofuran-2-ylmethylene)amino)phenyl)acetamide **(3)**

*
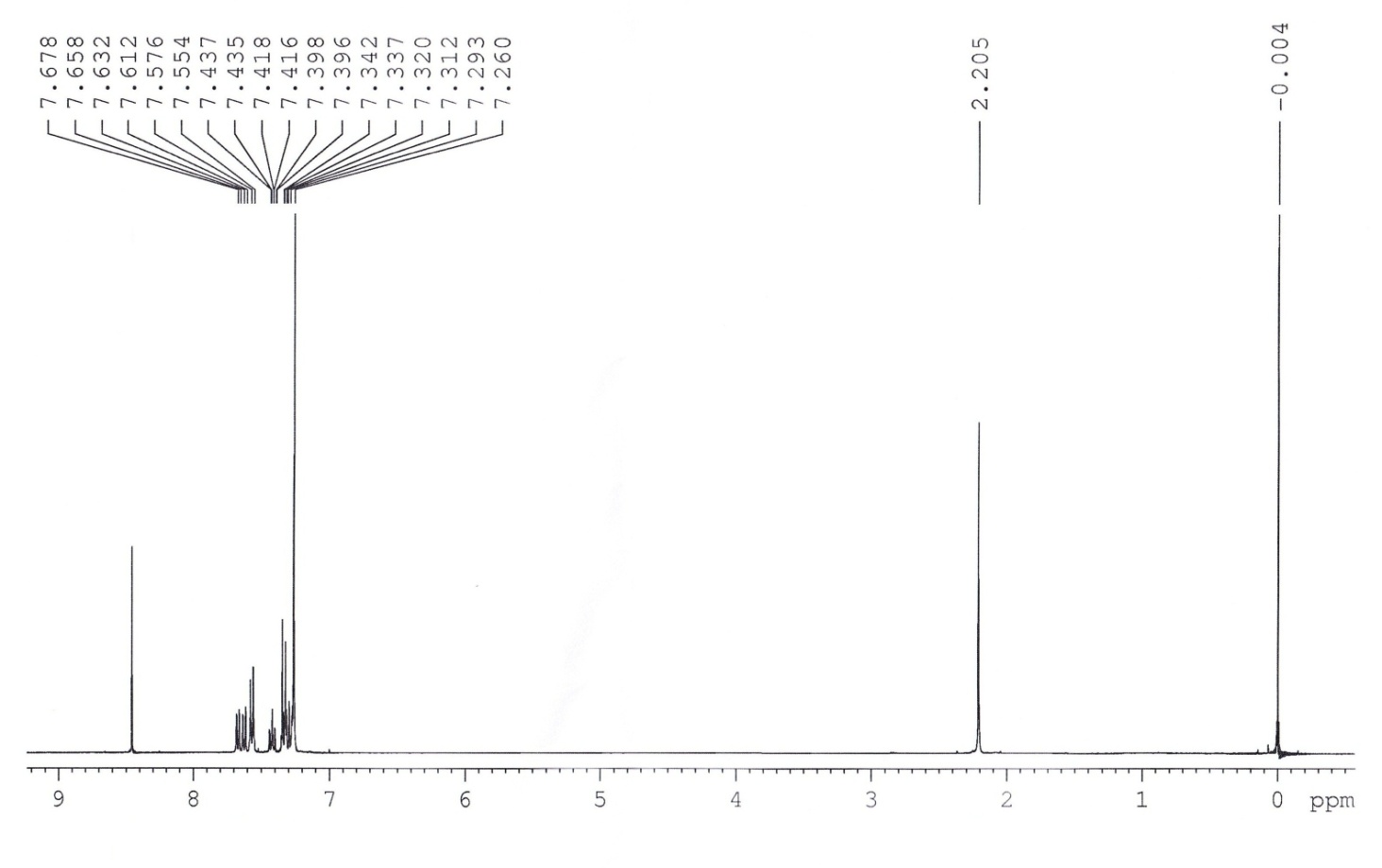
*

**S2:** ^1^H NMR spectrum of *N*-acetyl-*N*-(4-((benzofuran-2-ylmethylene)amino)phenyl)-2,5-difluoro benzamide **(5a)**

**
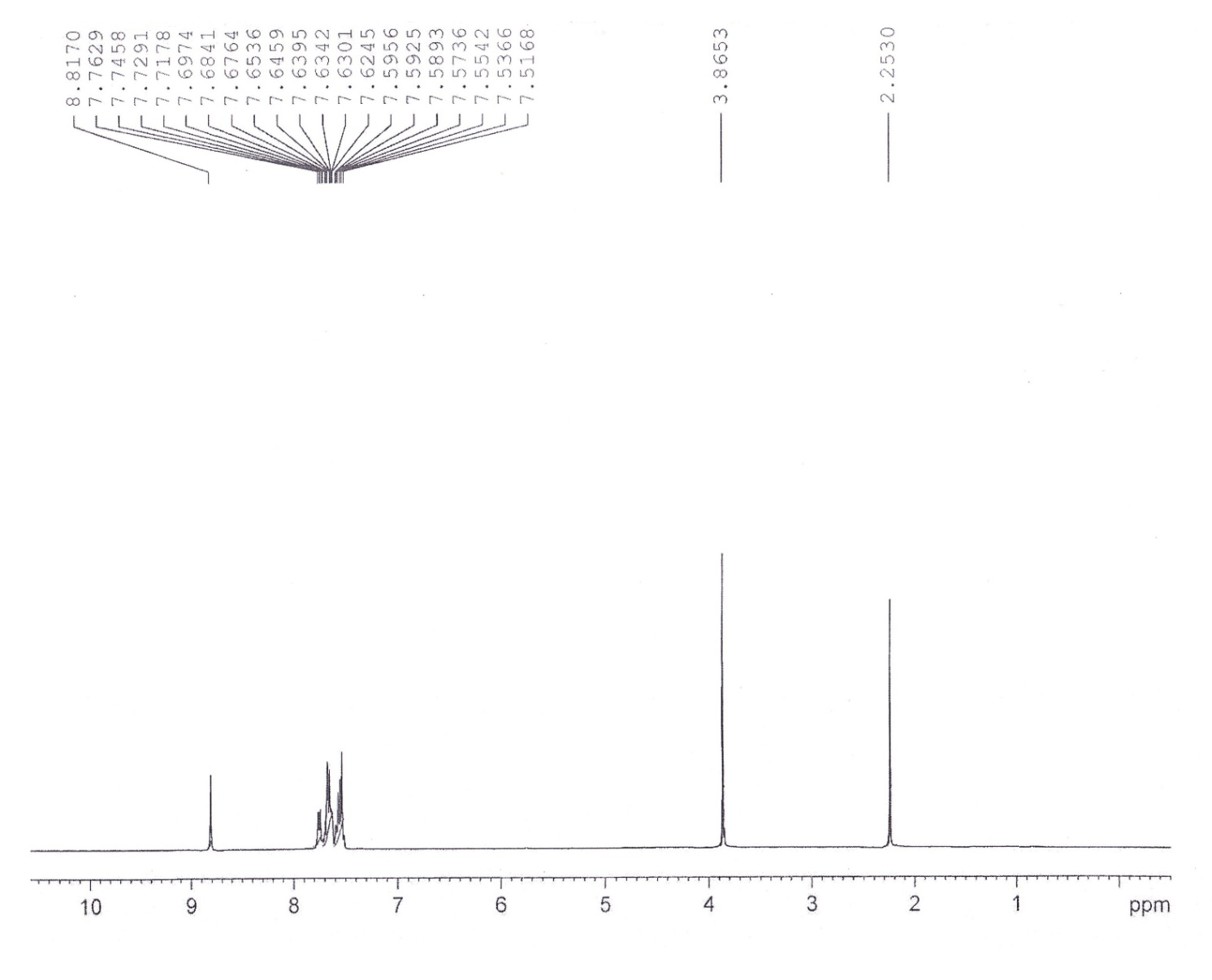
**

**S3:** ^1^H NMR spectrum of *N*-acetyl-*N* -(4-(benzofuran-2-ylmethyleneamino)phenyl)-4-methoxy benzamide **(5i)**

**
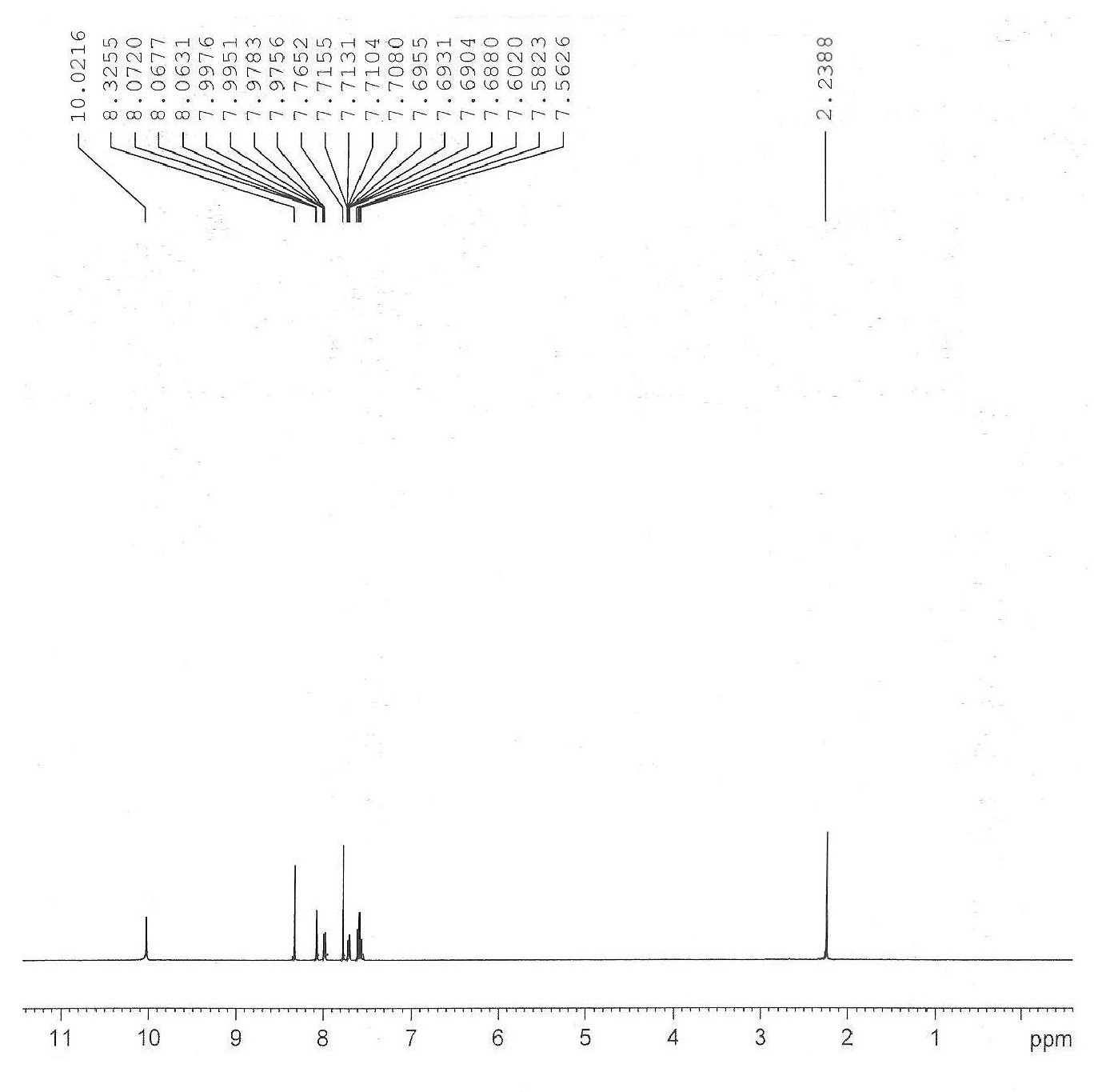
**

**S4:** ^1^H NMR spectrum of *N*-acetyl-*N* -(4-((benzofuran-2-ylmethylene)amino)phenyl)-4-hydroxy benzamide **(5j)**

**
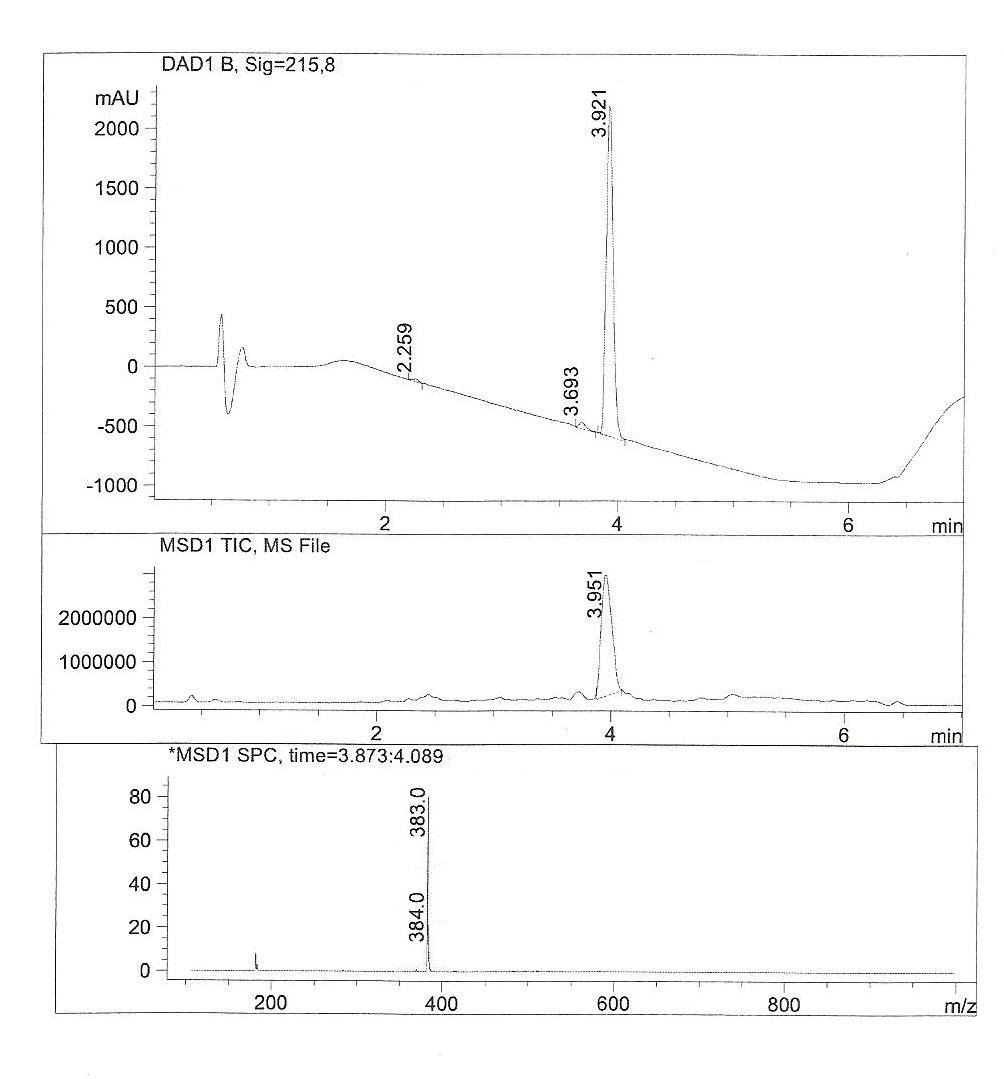
**

**S5:** Mass spectrum of *N*-acetyl-*N* -(4-((benzofuran-2-ylmethylene)amino)phenyl)benzamide **(5c)**

**
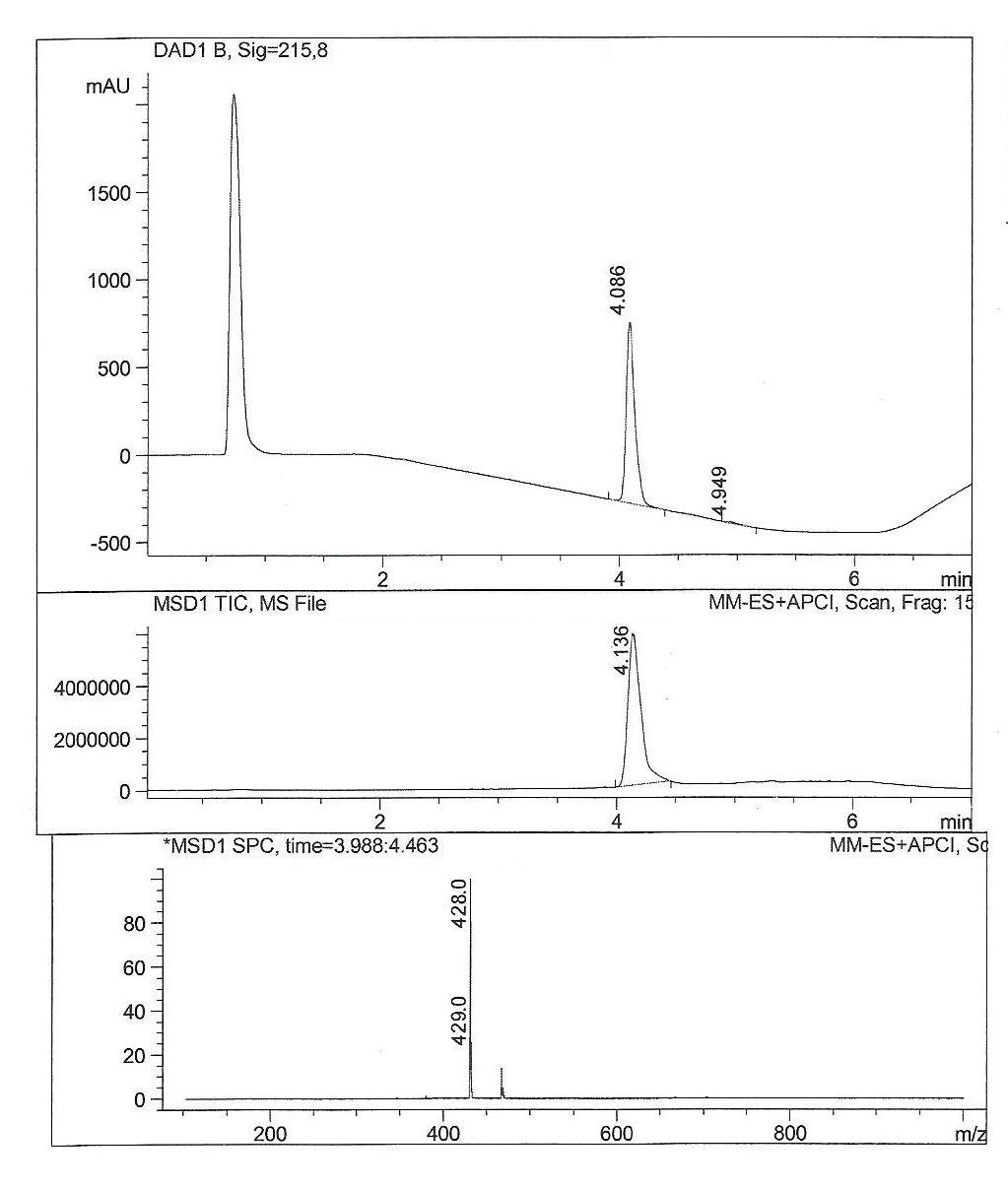
**

**S6:** Mass spectrum of *N*-acetyl-*N*-(4-((benzofuran-2-ylmethylene)amino)phenyl)-4-nitro benzamide **(5e)**

**S7:** IR spectrum of *N*-acetyl-*N*-(4-((benzofuran-2-ylmethylene)amino)phenyl)-3,5-difluoro benzamide **(5h)**

**S8:** IR spectrum of *N*-acetyl-*N*-(4-((benzofuran-2-ylmethylene)amino)phenyl)-4-hydroxy benzamide **(5j)**
